# Anaesthetic Management of Major Abdominal Oncosurgery in a Patient With an Automated Implantable Cardioverter-Defibrillator (AICD): A Case Report

**DOI:** 10.7759/cureus.36287

**Published:** 2023-03-17

**Authors:** Heena Saini, Suparna Barman, Jyotsna Goswami

**Affiliations:** 1 Anesthesiology, Tata Medical Center, Kolkata, IND; 2 Anesthesiology, All India Institute of Medical Sciences, Vijaypur, IND

**Keywords:** anaesthetic considerations, oncosurgery, low ejection fraction, aicd, ishemic cardiomyopathy

## Abstract

Ischaemic cardiomyopathy with low ejection fraction (EF) poses a perioperative challenge to the anesthetist due to the risk of hemodynamic instability, cardiovascular collapse, and heart failure. More so when a patient has an Automated Implantable Cardiovertor Defibrillator (AICD) in situ. We report the anesthetic management of a patient with ischaemic cardiomyopathy with an EF of 20% and AICD in situ posted for open right hemicolectomy. Dynamic hemodynamic monitoring with preparedness to manage fluid shifts, hemodynamic fluctuations, and adequate pain management is essential to successful anesthetic management in patients with an AICD, where programming is not possible.

## Introduction

Perioperative management of patients with dilated cardiomyopathy is a challenge to anesthesiologists due to poor left systolic function, ventricular enlargement, the risk of malignant arrhythmias, and sudden cardiac death [1]. Such cardiomyopathy may be ischemic or non-ischemic, with the former being related to atherosclerosis and ischemic heart disease [2]. In situ automated implantable cardioverter-defibrillators (AICDs) pose an additional challenge as they are in a restricted cardiac output state with an uncertain response to arrhythmias. The implanted devices play a vital role in managing life-threatening conditions such as severe bradycardia, pulseless ventricular tachycardia, and ventricular fibrillation.

The important considerations in this regard are AICD device interrogation, prior reprogramming, and perioperative interference due to electrolyte disturbance and electromagnetic interference leading to device failure and hemodynamic compromise. A magnet can be used when reprogramming is not possible; when a magnet is applied to an AICD, the anti-tachycardia function is suspended, but pacing capability is maintained. However, the procedure must be performed by knowledgeable technicians or doctors with sufficient experience in handling the resultant asynchronous rhythm [3]. Here, we report the anesthetic management of a patient with ischemic cardiomyopathy with an ejection fraction (EF) of 20% and an in situ AICD posted for abdominal oncosurgery where reprogramming was not possible. An institutional review board waiver and patient consent were obtained for the case report.

## Case presentation

**Figure 1:**
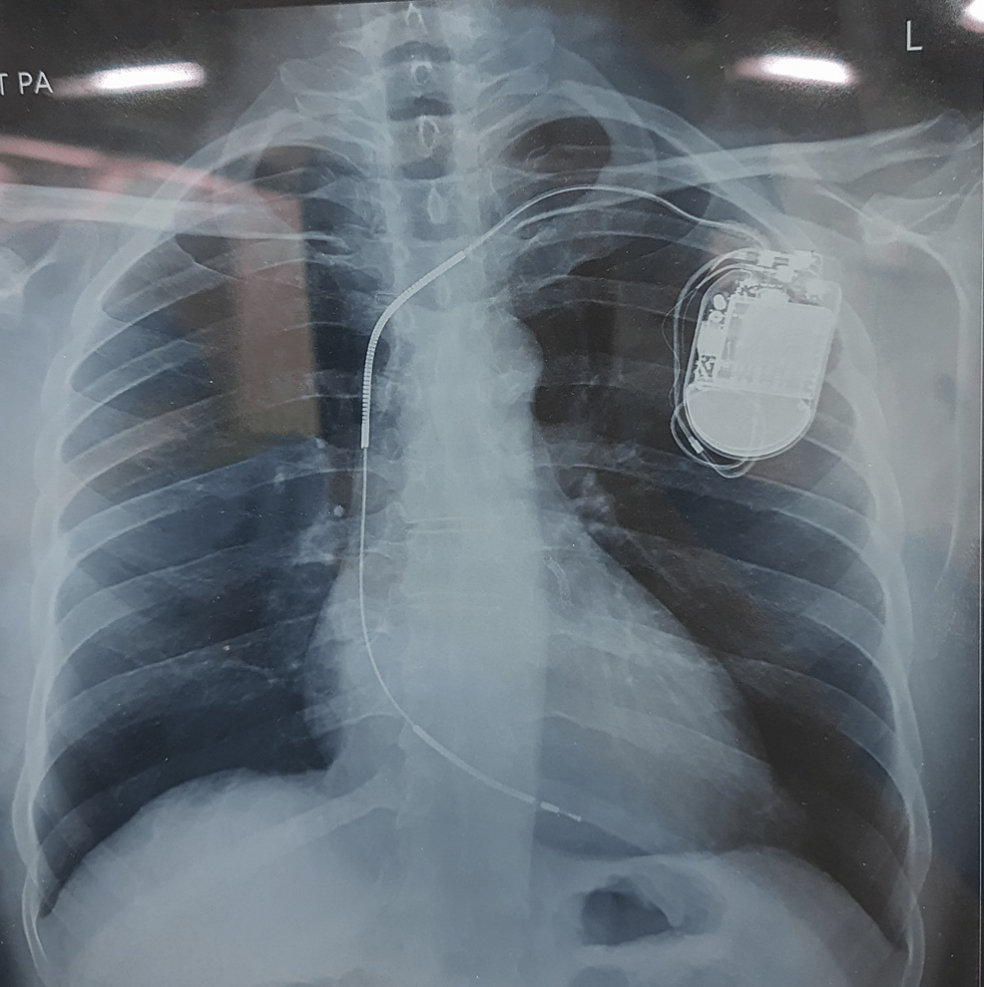
Chest X-Ray of the patient with AICD in situ

A 65-year-old male patient with ileocecal adenocarcinoma was posted for an open right hemicolectomy. He had a known case of hypertension that was controlled by multiple medications, mild bronchial asthma, and a past history of anterior wall myocardial infarction in 2007 that was treated with percutaneous transluminal coronary angioplasty with a drug-eluting stent placed in the left anterior descending artery. His severe ischemic cardiomyopathy was treated with an AICD (DURATA™ 7120-65cm, St Jude Medical, Sylmar, CA, USA) that was back-programmed (three years) in ventricular demand pacing mode, with sensing, pacing, and inhibiting of the right ventricle with both anti-tachycardia function and bradycardia pacing (VVI Mode) (Figure 1). His current medications were Tab Torasemide 10 mg+Tab eplerenone 25 mg once daily. After breakfast (ABF), Tab Ramipril 2.5 mg once daily ABF, Tab Carvedilol 6.25 mg BD, Tab Ivabradine 5mg, Tab Eplerenone 25mg ABF, Tab Rosuvastatin 10 mg+Tab Aspirin 75 mg once daily at bedtime. Patient also had a history of mild persistent bronchial asthma on inhaler (salmeterol and fluticasone combination). He also had a bifascicular block and 2D echocardiography showing moderate mitral regurgitation, a dilated left ventricle with generalized hypokinesia, and severe systolic and diastolic dysfunction with an EF of 20%.

Emergency drugs and external pacing devices, including a transcutaneous pacing device and a defibrillator, were made available. A magnet was applied over the AICD to change its mode to DOO 70 beats/min, an asynchronous dual-chamber pacing mode, and also to suspend anti-tachycardia functions without affecting the pacing mode. An epidural catheter using an 18 g Tuohy needle was inserted at the L1-L2 level with the patient in a sitting position. Arterial and central venous access was secured under a 2% lignocaine topical before the induction of anesthesia. The arterial cannula was connected to a cardiac output monitor (EV 1000 monitor) through a FloTrac transducer set (Edward Lifesciences, USA) to guide perioperative fluid management and inotropes. Dobutamine (2.5-5 mcg/kg/min) and norepinephrine (0.4-0.8 mg/hour) was started and titrated to acceptable parameters. After preoxygenation, induction was done with sevoflurane inhalation and 100 mcg of intravenous (IV) fentanyl, and 1 mg of midazolam. A neuromuscular blockade was achieved with 50 mg of rocuronium (inj.) to facilitate tracheal intubation. Maintenance was done with sevoflurane in an oxygen/air mixture and a continuous infusion of cisatracurium. Normothermia was maintained with the use of a convective air warmer.

Intraoperative fluid management was guided by various hemodynamic parameters such as stroke volume, cardiac output, stroke volume index (SVI), stroke volume variation (SVV), and systemic vascular resistance to maintain the following parameters: mean arterial pressure > 65 mmHg, SVV < 13%, SVI > 35 mL/m2/beat, and urine output = 0.5-1 mL/kg/h. The patient’s intraoperative arterial blood gas (ABG) analysis was acceptable. Intraoperative analgesia of paracetamol infusion (1 gm) and a bolus dose of epidural morphine (3 mg in 8 mL of normal saline) were used to avoid any immediate hypotension induced by local anesthetic agents. Diathermy using bipolar leads was used throughout the procedure. Blood loss was estimated at 100 mL, and the patient received 800 mL of IV fluids (crystalloid).

At the end of the four-hour surgery, tracheal extubation was performed after adequate reversal of the neuromuscular blockade, and the patient was transferred to the ICU for further monitoring. The patient’s AICD was restored to its preoperative parameters after the four-hour surgery. Postoperative pain was managed by an epidural infusion of 0.1% ropivacaine and 2 mcg/mL of fentanyl at a rate of 4-8 mL/h. The postoperative period was uneventful, and the patient was discharged on the sixth postoperative day.

## Discussion

The anesthetic management of patients and cardiomyopathy with low EF is challenging, with the goals of minimizing the negative inotropic and vasodilatory effects of anesthetic drugs and central neuraxial blocks, maintaining adequate preload and afterload, and controlling arrhythmias and hypotension [4]. In this case, we secured invasive monitoring access before induction for the early diagnosis of hemodynamic changes and prompt intervention. Continuous ECG (set at “diagnostic”), pulse oximetry, and continuous arterial pressure waveform monitoring are necessary for beat-to-beat assessment, for proper correlation of artifacts and electromechanical interference (EMI), and to identify pulseless electrical activity. Narcotics-based slow induction and balanced anesthetic techniques provide hemodynamic stability compared to routine induction agents like propofol. As a prolonged circulation time may delay the response of induction agents, adequate time should be allowed before additional doses of induction agents are used [5]. Inotropic support is commonly required, with inodilators usually used with vasoconstrictor agents to counteract the vasodilatory effects of the latter [6]. The titrated infusion of such drugs, started prior to induction, along with the monitoring of dynamic hemodynamic variables, resulted in an uneventful intraoperative course in our patient.

During the preanesthetic evaluation, the type, manufacturer, and programming mode of the AICD device should be noted from the patient’s identification card. In emergency situations, postoperative scarring on the pectoral/subclavicular region or a chest X-ray can be used to identify AICD electrodes. It is also necessary to assess pacemaker dependency from medical records or 12-lead electrocardiography. Although anti-bradycardia pacing is needed in around 25% of patients, the primary function of AICD devices is anti-tachycardia pacing and defibrillation. Device reprogramming before surgery is essential in patients who are vitally dependent on anti-bradycardia pacing, as the magnet does not affect anti-bradycardia function. If the AICD is reprogrammed (detection switched off) or a magnet is used to deactivate the therapeutic mode, as in our patient, then the treatment of malignant arrhythmias requires either the removal of the magnet to restore device function or the use of external defibrillation with the magnet [7].

EMI can trigger dysrhythmias and even deliver an inappropriate defibrillator shock in AICD patients. Therefore, we used the following precautionary measures to restrain EMI: (1) using a bipolar cautery device, (2) placing the cautery plate away from the site of device implantation, (3) limiting cautery use to the shortest possible duration, (4) using irregular bursts, and (5) using more “cutting” than “coagulating” current [8]. Arrhythmias can be managed with lidocaine, amiodarone, diltiazem, or cardioversion/defibrillation. In patients with dilated cardiomyopathy, goal-directed fluid therapy is useful for maintaining an optimal fluid balance to avoid both fluid overload and hypotension [9]. The intraoperative use of epidural morphine instead of local anesthetics can maintain hemodynamic stability [10].

## Conclusions

Ischemic cardiomyopathy with low EF is a real challenge to anesthetists, with risks of hemodynamic instability, cardiovascular collapse, and heart failure, especially in patients with in situ AICDs. Device interrogation and reprogramming of the in situ AICD are essential before the patient is taken up for surgery. Rigorous hemodynamic monitoring, prompt intervention in the case of any eventuality, maintaining fluid homeostasis, and good pain management are essential for uneventful anesthetic management.
